# ABCA7—A Member of the ABC Transporter Family in Healthy and Ailing Brain

**DOI:** 10.3390/brainsci10020121

**Published:** 2020-02-22

**Authors:** Alexei A. Surguchev, Andrei Surguchov

**Affiliations:** 1 Department of Surgery, Section of Otolaryngology, Yale School of Medicine, Yale University, New Haven, CT 06510, USA; alexei.surguchev@yale.edu; 2 Department of Neurology, University of Kansas Medical Center, Kansas City, KS 66160, USA

**Keywords:** Alzheimer’s disease, ATP-binding cassette, ABCA7, amyloid-β, amyloid precursor protein, presenilin

## Abstract

Identification of genetic markers of a human disease, which is generally sporadic, may become an essential tool for the investigation of its molecular mechanisms. The role of ABCA7 in Alzheimer’s disease (AD) was discovered less than ten years ago when meta-analyses provided evidence that rs3764650 is a new AD susceptibility locus. Recent research advances in this locus and new evidence regarding ABCA7 contribution to the AD pathogenesis brought a new understanding of the underlying mechanisms of this disorder. An interesting, up-to-date review article "ABCA7 and Pathogenic Pathways of Alzheimer’s Disease" by Aikawa et al. (2018), outlines the ABCA7 role in AD and summarizes new findings in this exciting area. ABC transporters or ATP-binding cassette transporters are a superfamily of proteins belonging to a cell transport system. Currently, members of the family are the focus of attention because of their central role in drug pharmacokinetics. Two recent findings are the reason why much attention is drawn to the ABCA7 family. First, is the biochemical data showing a role of ABCA7 in amyloid pathology. Second, genetic data identifying ABCA7 gene variants as loci responsible for the late-onset AD. These results point to the ABCA7 as a significant new contributor to the pathogenesis of AD.

## 1. Alzheimer’s Disease (AD) is a Devastating Neurodegenerative Disease 

Currently, an estimated 5.8 million Americans have AD, and this number is growing quickly. Two hundred thousand individuals under age 65 have younger-onset AD [1]. A key feature of AD is a buildup of extracellular amyloid-β (Aβ) and corresponding plaques within the brain. This accumulation occurs because of the altered balance between Aβ production and clearance. Another important characteristic of AD is the accumulation of hyperphosphorylated Tau protein. This buildup leads to an increase in neurofibrillary tangles (NFTs). Accumulation of NFTs represented by bundles of filamentous protein occurs most often in the cytoplasm of neurons. Elevated levels of NFTs, neurotoxic Aβ peptides, and loss of neurons and synapses, result in brain atrophy. These are the main factors in the progression of AD, which can be classified as a conformational disease [2] because of the roles of naturally unfolded prone to aggregation proteins in its development [3,4]. Currently, basic researchers and pharmaceutical companies have put an enormous amount of effort and funds toward finding novel targets for pharmacological interventions for AD.

## 2. Contribution of Genetic Factors to AD Pathogenesis

The majority of AD cases are late-onset and sporadic. However, investigation of genetic forms of AD and identification of susceptibility loci for late-onset Alzheimer’s disease (LOAD) is an important approach raising hope for finding new mechanisms and new targets in AD pathogenesis. Although genetic forms of AD are relatively rare, their investigation helps discover detailed mechanisms of this disorder [5]. Researchers use various approaches to detect the genetic variants contributing to disease traits with complex inheritance. The ε4 allele of apolipoprotein E (ApoE), remains the most significant sequence variant affecting the risk of late-onset AD [6]. Genetic studies revealed other major risk factors that markedly affect the risk of developing AD. The majority of them are rare variants in the following genes: Amyloid precursor protein (APP), presenilin 1 (PSEN1), and presenilin 2 (PSEN2) [5]. Recently, a rare susceptibility variant in TREM2 was discovered [7,8]. Lambert et al. (2013) [9] performed a meta-analysis of GWAS in European ancestry and discovered a novel susceptibility variant rs4147929 in an intron of the ABCA7 gene. However, the existing genetic data could not explain all phenotypic forms of AD, and a large portion of the genetic risk for AD remains unexplained.

## 3. ABCA7 and AD

The recent biochemical and genetic data point to ABCA7 (ATP-binding cassette sub-family A member 7) as a new contributor to AD pathogenesis [10]. Common variants of this gene were associated with the risk for LOAD [11,12]. Recent evidence describing the risk of gene variants of ABCA7 for AD development and its role in the AD pathogenesis are described in the Aikawa et al. review [10]. Genetic data pointing to the ABCA7 gene contribution to AD began to appear about ten years ago. An important finding indicating a contributing role of ABCA7 in AD development was the identification of the common single nucleotide polymorphism (SNP) variant rs3764650 in an ABCA7 intron. This marker is the susceptibility loci for LOAD [11] in Caucasian cohorts. Next, a missense variant associated with the risk for LOAD due to the G1527A substitution in ABCA7 was described [13]. Analysis of ABCA7 gene variants in different populations by exome sequencing, whole-genome sequencing, and targeted resequencing confirmed the conclusion about the role of ABCA7 in AD. These studies showed that some of the low-frequency variants (1%–5%) and rare variants (less than 1%) have significant associations with the risk for AD [14]. Based on the data about the loss-of-function of ABCA7, the three most probable mechanisms of its involvement in AD pathology are proposed. First, is the disturbance of microglial Aβ clearance. Second, is the accelerated APP processing and third, is the interference with the elimination of various brain debris during AD progression.

## 4. ABCA7 Structure and Functions 

Members of the family are built of multiple subunits, and one or two of them are usually associated with membrane ATPases. ABCA7 is a large protein containing 2146 amino acids with a molecular weight of 220 kDa. Its closest homolog is ABCA1, with 54% of sequence identity. These two proteins also share some functional similarity [10], but their transcription is regulated differently. ABCA7 is a ubiquitous protein with high expression in microglia. It is expressed in a tissue-specific manner as two variants. Type I cDNA is a full-length, whereas Type II is a shorter splicing variant. ABCA7 maintains intracellular lipid metabolism and regulates cellular homeostasis. It is involved in the efflux of cellular phospholipids, cholesterol, phosphatidylcholine, and other lipids. The family includes about fifty members subdivided into seven subfamilies. Functions of the members of this family are to perform and control the efflux of intracellular cholesterol and phospholipids. ABCA7, in addition to the functions of the mediator of lipid metabolism, also participates in the generation of immune and regulation of microglial responses to acute inflammatory challenges.

The high level of ABCA7 expression in cell culture increases the amounts of intracellular/cell surface ceramide and intracellular phosphatidylserine, causing cell cycle arrest. The variety of processes in which ABCA7 is involved is amazing. In addition to being a modulator of several biochemical pathways, ABCA7 regulates phagocytic activity in microglia. This activity may play an important role in AD pathogenesis. In particular, ABCA7 is involved in phagocytosis of Aβ aggregates and, therefore, participates in Aβ elimination in the brain.

Furthermore, ABCA7 is an essential player in the regulation of APP processing. Several mechanisms pinpoint to this ABCA7 role. The most important one of them is via altered lipid profile. Indirect evidence supporting this point of view suggests that elevated levels of phospholipids, cholesterol, and phospholipids regulate APP processing. Due to the involvement of ABCA7 in many processes critically involved in AD pathogenesis, further investigation is necessary to uncover their relationship in more detail.
